# Supplementary material for: Rho enhancers play unexpectedly minor roles in Rhodopsin transcription and rod cell integrity
Source: Sci Rep. 2023 Aug 9;13:12899. doi: 10.1038/s41598-023-39979-6 (PMC10412641; doi:10.1038/s41598-023-39979-6)
Supplement: Supplementary file 1 — Supplementary Figures. [file 41598_2023_39979_MOESM1_ESM.pdf]

Rho enhancers play unexpectedly minor roles in *Rhodopsin* transcription and rod cell integrity

Chi Sun[1], Philip A. Ruzycki[1,2]* and Shiming Chen[1,3]*
[1]Department of Ophthalmology and Visual Sciences, [2]Department of Genetics, and [3]Department of Developmental Biology, Washington University, Saint Louis, Missouri, USA

*To whom the correspondence should be addressed.
Shiming Chen
660 South Euclid Avenue, MSC 8096-0006-06
St. Louis, MO 63110, USA
Tel. (314) 747-4350
chenshiming@wustl.edu

Philip Ruzycki
660 South Euclid Avenue, MSC 8096-0006-11
St. Louis, MO 63110, USA
Tel. (314) 747-5670
p.ruzycki@wustl.edu

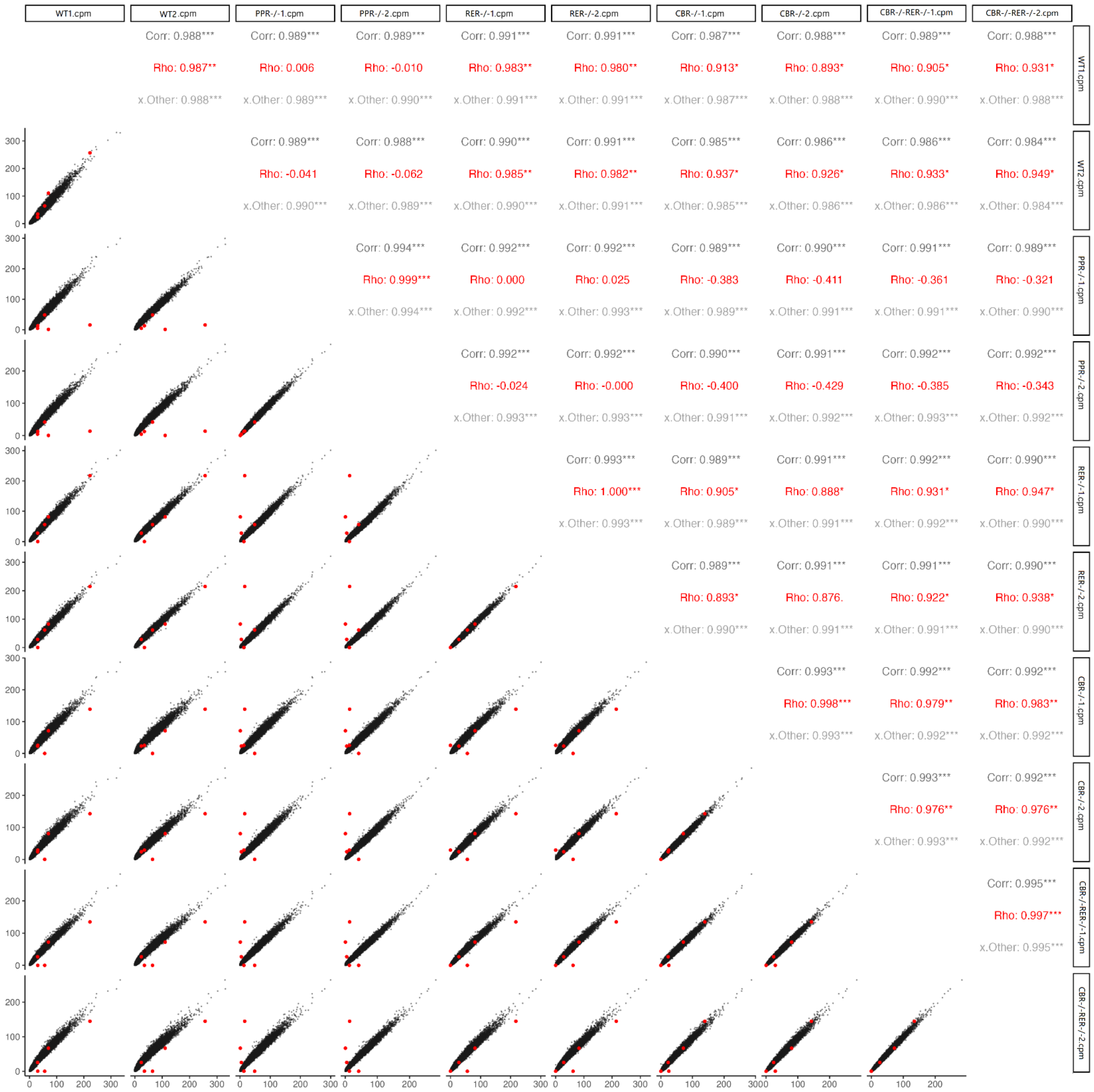

Supplemental Figure 1: **Scatterplots show high reproducibility of biological replicate ATAC-seq datasets.** Black dots represent quantification of all genome-wide ATAC-seq peaks, while red dots represent only the 5 peaks near Rho. Also presented are the Pearson correlation coefficient values (‘Corr’ = all data points, ‘Rho’ = 5 peaks near Rho, ‘Other’ = all non-Rho related peaks), supporting the very high correlation between biological replicates and genome wide high reproducibility across all samples.

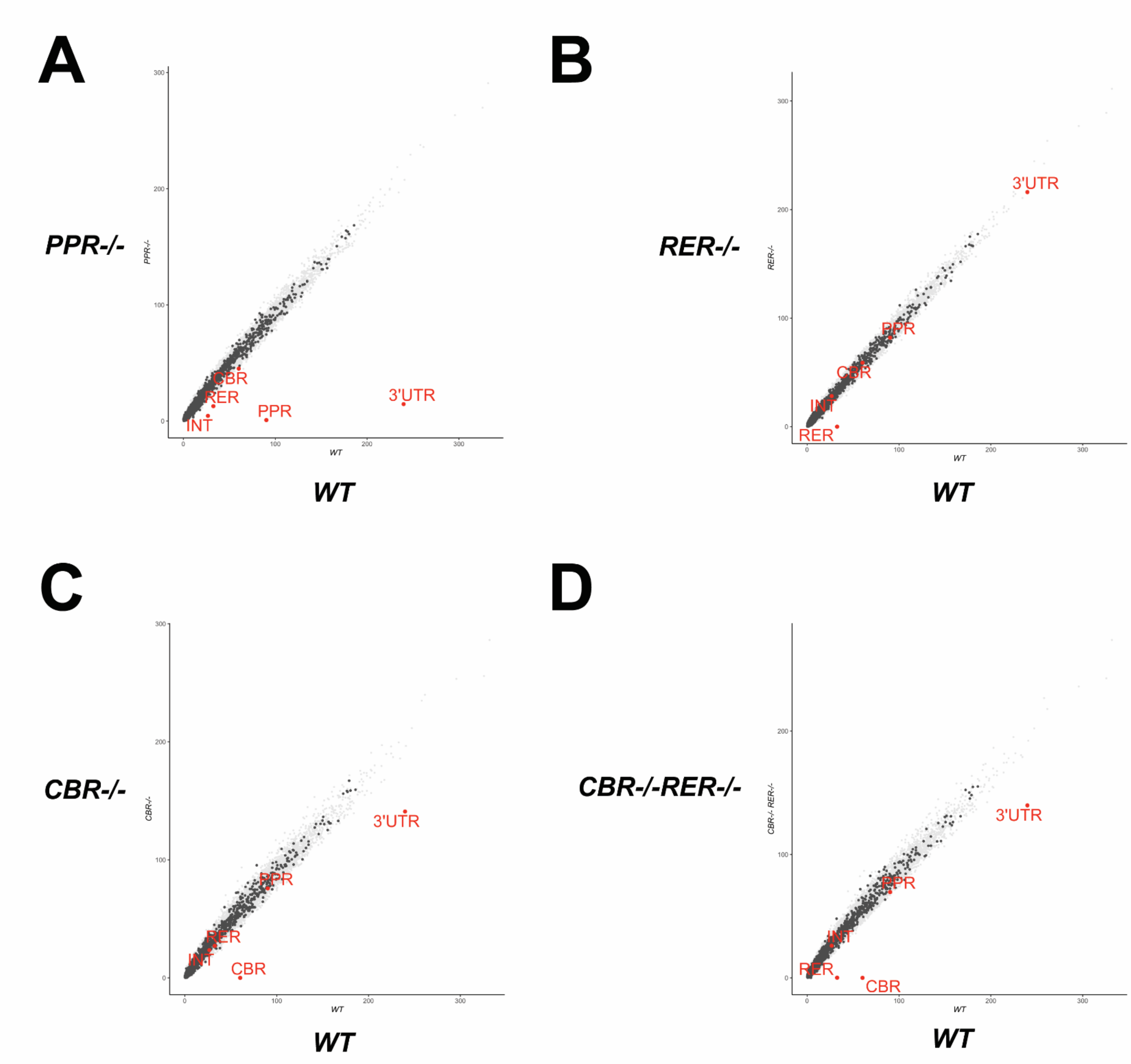

Supplemental Figure 2. **Scatterplot displays ATAC-seq signal changes at *Rho cis-regulatory regions* in the indicated mutants relative to the *WT* control**. *CBR*, *CRX-bound region 1*. *RER*, *Rhodopsin enhancer region*. *PPR*, *Rhodopsin proximal promoter region*. *INT*, *Rhodopsin Intragenic region*. *3' UTR*, *3' untranslated region*.

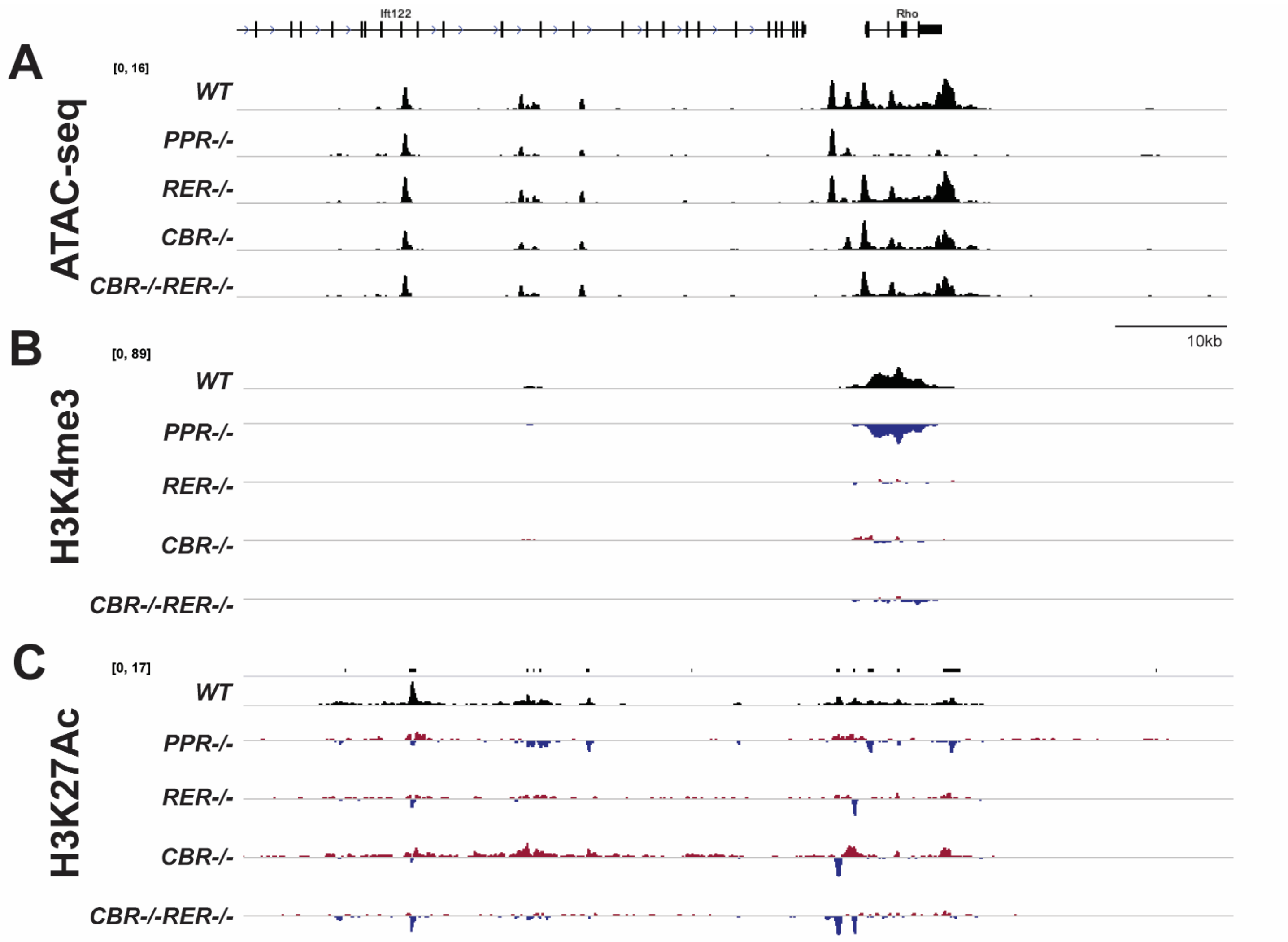

Supplemental Figure 3. Browser tracks display ATAC-seq (A), H3K4me3 (B), H3K27ac (C) signals within 50 kb upstream of *Rho* locus. A 10 kb bar is included for estimating size of the genomic region. The difference in H3K4me3 peaks ranges between -89 and 89. The difference in H3K27ac peaks ranges between -6.7 and 6.7. The four tracks below *WT* in panels (B) and (C) display increased (red) and decreased (blue) signals relative to the *WT* control in mutant retinas.

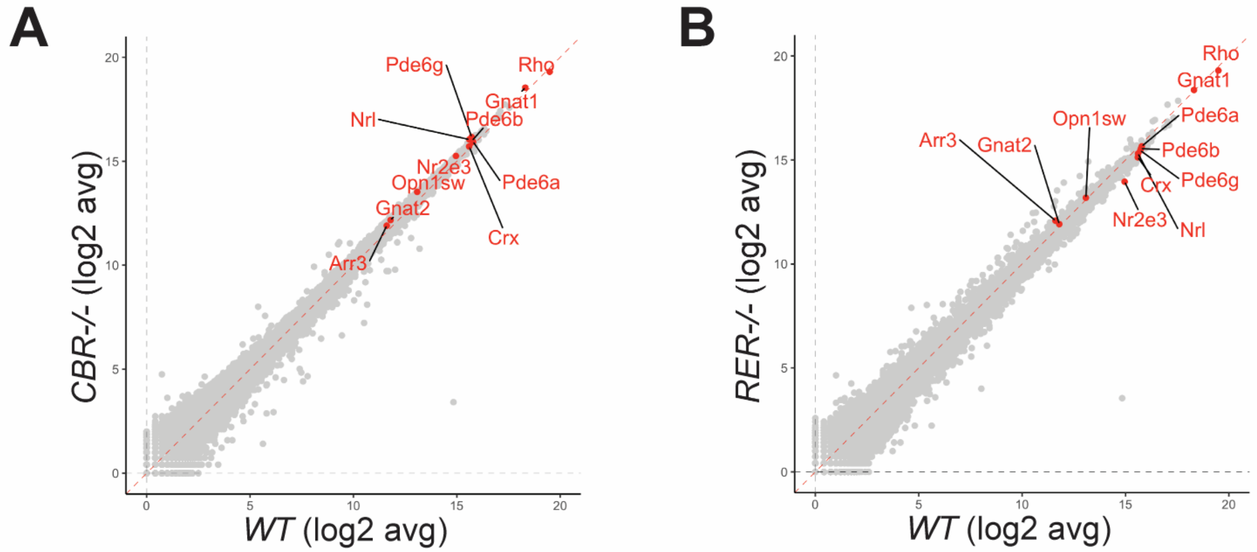

Supplemental Figure 4. RNA-seq analysis in P14 *CBR-/-* (A) and *RER-/-* (B) retinas. Each scatterplot displays the expression distribution of selected genes in the indicated mutant (Y-axis) relative to the *WT* control (X-axis).

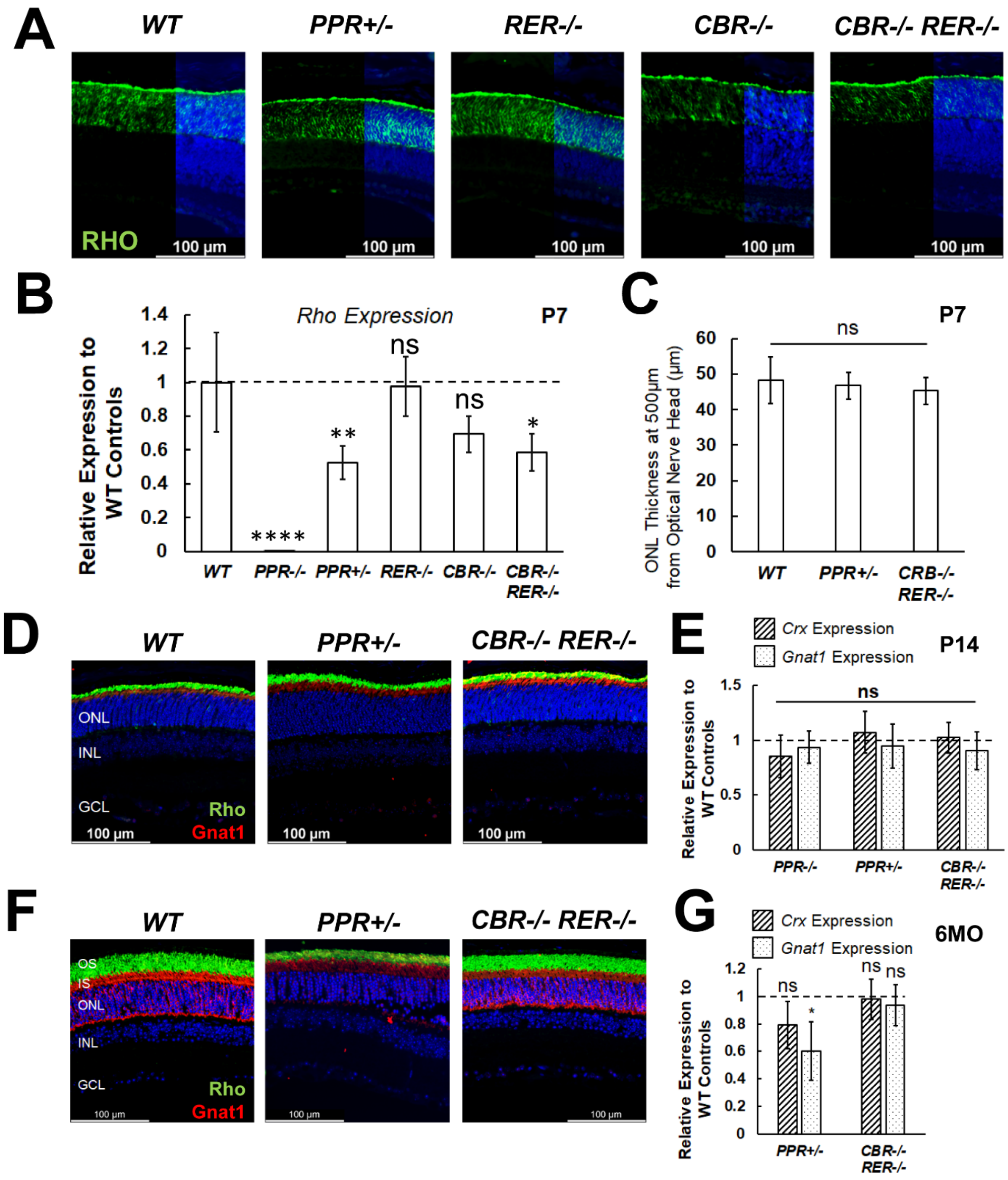

Supplemental Figure 5. Rod photoreceptor localization and morphology in mutant retinas. (**A**) Immunohistochemistry staining of RHO on retinal cross-sections of the indicated mice at P7. (**B**) qRT-PCR analysis of *Rho* expression in retinal samples of the indicated mice at P7. (**C**) ONL thickness in P7 *WT*, *PPR+/-*, and *CBR-/-RER-/-* retinas at 500µm from the optic nerve head. (**D, F**) Immunohistochemistry staining of RHO and GNAT1 on retinal cross-sections of the indicated mice at P14 (**D**) and 6MO (**F**). (**E, G**) qRT-PCR analysis of *Crx* and *Gnat1* expression in retinal samples of the indicated mice at P14 (**E**) and 6MO (**G**). Results are plotted as relative expression to the *WT* controls. Statistics is done by one-way ANOVA with Tukey's multiple comparisons. ns means not significant. Asterisks (*, **, ****) denotes $p \leq 0.05$, 0,01, 0.0001. Scale bar represents 100 µm for all image panels.

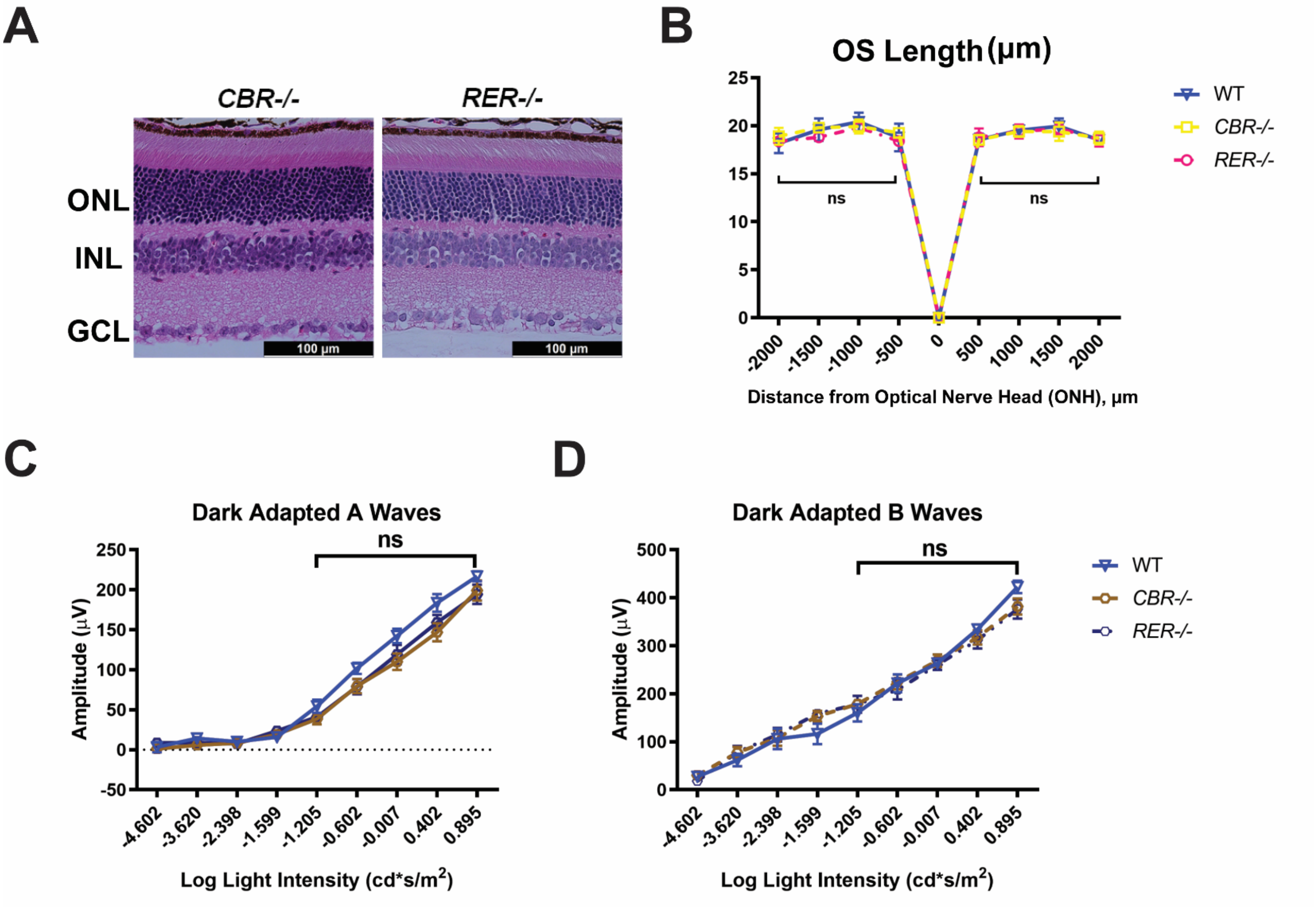

Supplemental Figure 6. Knockout of individual enhancers does not impact retinal morphology and function. (**A**) Hematoxylin and Eosin (H&E) cross-section staining of 6MO *CBR-/-*, and *RER-/-* retinas. ONL: outer nuclear layer; INL: inner nuclear layer; GCL: ganglion cell layer. Scale bar = 100 µm for all image panels. (**B**) OS thickness in 6MO *WT*, *CBR-/-*, and *RER-/-* retinas at various positions from the optic nerve head (ONH). Error bars represent mean (SD) (n ≥ 4). (**C, D**) Electroretinogram (ERG) analysis of 6MO *WT*, *CBR-/-*, and *RER-/-* mice, showing mean amplitudes (µV) of dark-adapted A-waves (**C**) and B-waves (**D**) at various stimulus light intensities. Error bars represent SEM (n ≥ 7). All statistics is done by comparing to *WT* control with two-way ANOVA with Tukey's multiple comparisons. ns means not significant.

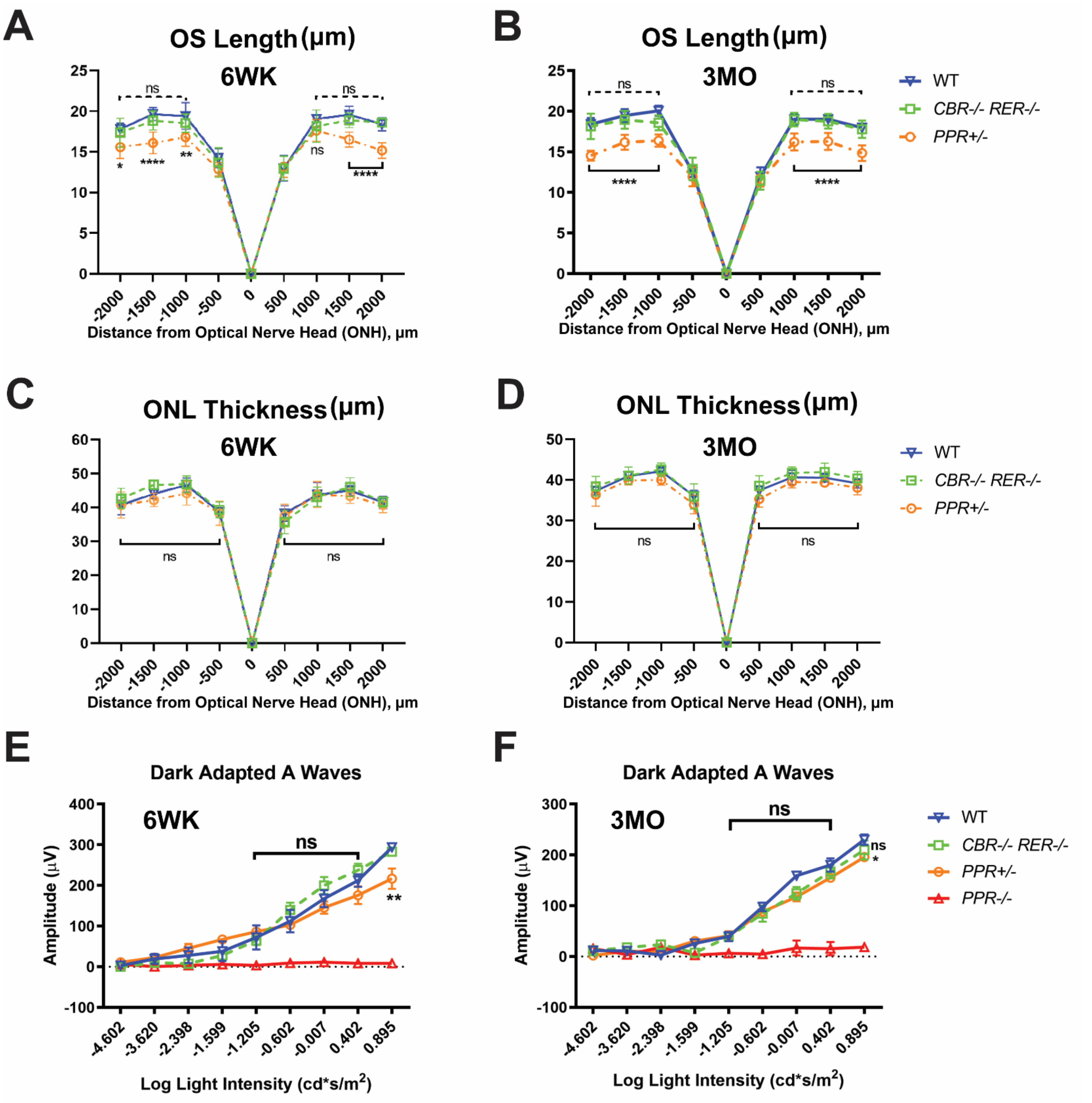

Supplemental Figure 7. **Knockout of both *Rho* enhancers (*CBR-/-RER-/-*) does not alter retinal morphology and function in young adults.** (**A** & **B**) OS thickness in 6WK (**A**) and 3MO (**B**) retinal samples of *WT* and mutants (n ≥ 4). Dashed black line indicates the statistical difference between *WT* and *CBR-/-RER-/-* samples, solid black line indicates the comparison between *WT* and *PPR+/-* samples. (**C** & **D**) ONL thickness in 6WK (**C**) and 3MO (**D**) retinal samples of *WT* and mutants (n ≥ 4). Solid black line indicates the statistical difference between *WT* and mutant samples. (**E** & **F**) Electroretinogram (ERG) analysis of dark-adapted A-waves for 6WK (**E**) and 3MO (**F**) samples of *WT* and mutants. Mean amplitudes (µV) are plotted against stimulus light intensity. Error bars represent SEM (n ≥ 7). All statistics is done by comparing to *WT* control with two-way ANOVA with Tukey's multiple comparisons. Asterisks (*, **, ****) denote $p \leq 0.05$, $p \leq 0.01$, $p \leq 0.0001$, respectively. ns means not significant.

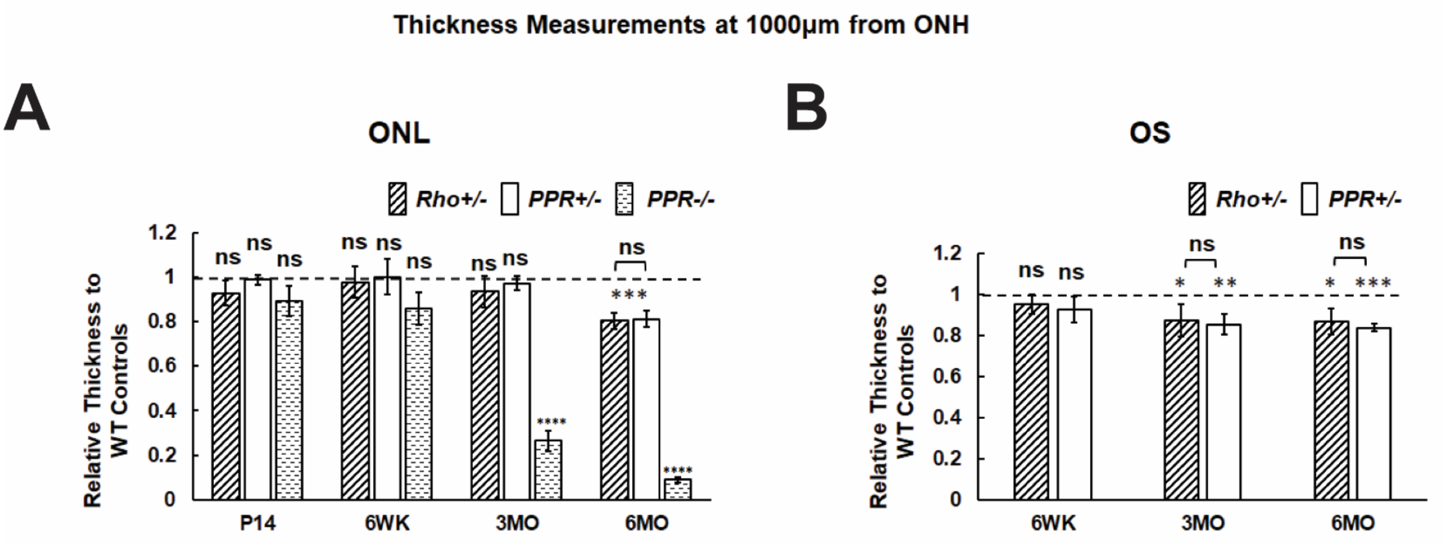

Supplemental Figure 8. Comparable analysis of ONL and OS thickness in *Rho PPR* and *Exon* knockout mutants. Bar graphs of ONL (**A**) and OS (**B**) thickness in *Rho+/-*, *PPR+/-*, *PPR-/-* retinas relative to *WT* control at different ages. Asterisks (*, **, ***, ****) denote p ≤ 0.05, p ≤ 0.01, p ≤ 0.001, p ≤ 0.0001, respectively by one-way ANOVA with Tukey's multiple comparisons. ns means not significant.
